# Optimization of Pulsed Laser Cladding for Reconditioning of Ni–Al–Bronze (NAB) Marine Propeller

**DOI:** 10.3390/ma18184301

**Published:** 2025-09-14

**Authors:** George Ciprian Iatan, Dan Cristian Cuculea, George Ardelean, Elena Manuela Stanciu, Alexandru Pascu

**Affiliations:** Materials Engineering and Welding Department, Transilvania University of Brasov, Eroilor Blvd., 29, 500036 Brasov, Romania; george.iatan@unitbv.ro (G.C.I.); dan.cuculea@unitbv.ro (D.C.C.); george.ardelean@unitbv.ro (G.A.); elena-manuela.stanciu@unitbv.ro (E.M.S.)

**Keywords:** laser cladding, nickel–aluminum bronze, marine propeller

## Abstract

The materials used in the marine environment are generally selected for their high performances in aggressive operational media. This is also the case for marine propellers, which are mainly manufactured from cast nickel–aluminum bronze (NAB), due to their favorable mechanical properties and corrosion resistance. This study is focused on maximizing the efficiency of pulsed laser cladding through coaxial powder feeding, aiming to develop it as a sustainable reconditioning method for NAB propellers. A pulsed-wave laser (Trumpf TruPulse 556) and a cladding head (Precitec WC 50) were used for cladding of CuNi-alloyed powder on an NAB substrate. One of the main challenges was the high reflectivity of the copper matrix, present in both the base material of the propeller and in the powder, which significantly reduces laser energy absorption. However, good-quality cladded layers were obtained by optimizing the process cladding parameters. The coatings were characterized by optical and scanning electron microscopy. Microhardness values indicated transition regions within the coating layer. The results demonstrate that laser cladding with pulsed lasers is an effective and promising surface engineering method for reconditioning of damaged marine propellers. The obtained results create a path for future research aimed at extending the service life of copper-based marine components.

## 1. Introduction

Since the development of the marine propeller concept in the 19th and 20th centuries, various modern approaches have emerged in terms of materials due to current challenges related to acoustic performance, corrosion and wear resistance, production costs, and material source availability [1]. Techniques like conventional MIG welding [2] were used to improve the cavitation erosion of the coatings, and by using high-speed laser cladding [3], a deposition efficiency of 156.79 cm^2^/min was accomplished. Further improvements were reported by exploring the potential of novel materials such as composites [4], polymers, and carbon fiber mixtures aiming to counter industry drawbacks. The flexibility enhancement of ship propellers was attempted by Neser G. et al. [5] using high-density polyethylene/long carbon fiber, and Zhang [6] reduced the propeller noise using an innovative geometric design. Glass fiber-reinforced composites [7] and carbon fiber composite propellers [8] have been intensively studied as possible replacement materials for nickel or copper alloys. However, it is reported that most propellers are still manufactured from bronze matrix alloys [9]. Known as nickel–aluminum bronze (NAB), this material has been proven to be among the most reliable copper alloys exposed to the marine environment due to its excellent corrosion resistance and good mechanical behavior [10]. Despite those advantages, the complex phase structure of NAB and the work conditions of propellers make it vulnerable by altering certain phases over time. While some phases are more resistant to corrosion mechanisms than others, the overall integrity of the marine propeller is affected gradually as time passes [11].

When assessing damage to marine propellers, the approach becomes complex due to the variety of stakeholders involved. As analyzed by Dev et al. [12], maintenance of the ship propeller is mainly related to periodical docking of the vessel. For a better understanding of the recurrence of the bottom examination, the related rules from main Classification Societies from IACS, mainly LR [13], DNV-GL [14], RINA [15] and ClassNK [16], were reviewed. Thus, the propeller of a merchant vessel is subject to inspection and examination at least once in 36 months according to related rules [13,14,15,16]. Hence, it is difficult for the interested parties to notice whether the propeller suffered any damage within this interval. This is why a reliable technology for addressing repairs of the propeller is important.

In the last few decades, procedures involving welding, casting or thermal spray have been applied to repair worn areas of marine components.

Yet, there are studies indicating that these procedures might represent a risk in terms of worker safety, environmental protection and final quality of the product [17]. Repair works for marine propellers are a demanding task requiring special work conditions, specialized personnel with extensive experience and outdated technologies. At the same time, new methods have become available for repairing the NAB components.

In the context of emerging technologies entailing a high degree of automation, integration of IoT and advanced robotic systems assisted by artificial intelligence and customized software, optimized results can be obtained faster with longer-term persistence. Such an example is represented by laser cladding technologies.

Recent studies have shown increased interest in identification of novel methods for reconditioning and optimization of NAB marine propellers. For example, Li, Z. et al. [18] employed a 5 kW fiber laser for coating TaC/Co-based preplaced powders, resulting in coatings with uniform distribution, refined microstructure and increased microhardness of the NAB sample. A fiber laser was also used by An et al. [19] for cladding CuNi powders. The research consisted of fabrication of a corrosion-resistant coating by extreme high-speed laser cladding, resulting in improved characteristics. Furthermore, the influence of the heat input on the cladding geometry is emphasized. Although the results are positive, the authors suggest laser remelting as an additional measure for improvement of the corrosion resistance of CuNi coating.

Bourahima et al. [20] studied how process parameters affect the cladding geometry and the adhesion. A 4 kW continuous laser was used for coating Ni-based powder on a Cu-Ni-Al base material. They used the ANOVA method to optimize the process parameters while respecting the predefined geometrical dimensions and the minimum power required to ensure quality clad/substrate bonding. One important challenge when dealing with laser cladding of complex alloys is the prevention of crack occurrence. In this context, a comprehensive approach to crack types and factors influencing their manifestation is presented in detail by Li, M. et al. [21]. Several methods such as adding alloying elements, using transition layers, optimization of process parameters, heat treatments, auxiliary fields like magnetic fields or ultrasonic vibration and stress prognosis through numerical analysis prior to experimental work are some of the countermeasures suggested by the authors. The issue of cracking is detailed in other studies. Thus, it has been reported that stress corrosion cracking, generated by the interference in the material, environment and stress strain relationship, is a source of early failure of materials. Furthermore, although the corrosion performance of CuNi alloys is proven due to the Cu_2_O oxide layer formation on the surface, the presence of ions such as S2 exposes the material to stress corrosion cracking. As a countermeasure to this, corrosion inhibitors like glycine and benzotriazole are proposed [22]. Other research teams decreased crack susceptibility in laser cladding of Ni alloys by ameliorating the thermal expansion coefficient between the cladded material and substrate, through handling thermophysical parameters [22,23].

Pulsed lasers have also shown potential in this area. In this regard, Ebrahimzadeh et al. [24] demonstrated that optimization of process parameters by using a rectangular shaped pulse led to a decrease in the solidification rate, thus reducing crack occurrence in the brittle temperature range. In accordance with their obtained results, a pulse duration of about 10 ms and frequency of 1 Hz enabled elimination of crack formations. Furthermore, augmented preheating temperature was noticed as a reason for hot cracking. Crack-free coated layers were also obtained by Khorram et al. [25] through a dilution ratio lower than 28%, which was ensured by an increased laser speed and reducing laser frequency and pulse width. In terms of advancements in corrosion-resistance treatments for materials exposed to hard environments with CuNi matrix layers, Pingale et al. [26] present several steps and methods for electrodeposition, as an alternative to laser cladding. However, although the results are promising, the methods for this technique limit the coated layer to thicknesses up to 200 µm. A valuable state-of-the-art review of the laser cladding techniques where copper alloys are employed is presented by the research team of Jin Ling [1]. The paper presents the key experimental results in the field of laser cladding on copper alloys emphasizing ongoing challenges related to the low absorption of copper and the difficulty of achieving strong, defect-free metallurgical bonds.

This study aims to advance the field of laser cladding by using a modern pulsed laser-based approach to repair marine propellers. Although damages affecting the ship propellers have been widely investigated and multiple repair methods were proposed, few studies have focused on reconditioning using pulsed laser cladding. Li et al. [18] and An et al. [19] obtained good results but relied on continuous or high-speed laser processing. The pulsed laser offers significant advantages like reduced thermal stress and controlled dilution, being suitable for reconditioning applications.

The present research aims to investigate a new technique using pulsed laser cladding as a high-performance solution for repairing the NAB-based material in the field of marine propellers with improved mechanical and corrosion performance.

## 2. Materials and Methods

**Figure 1:**
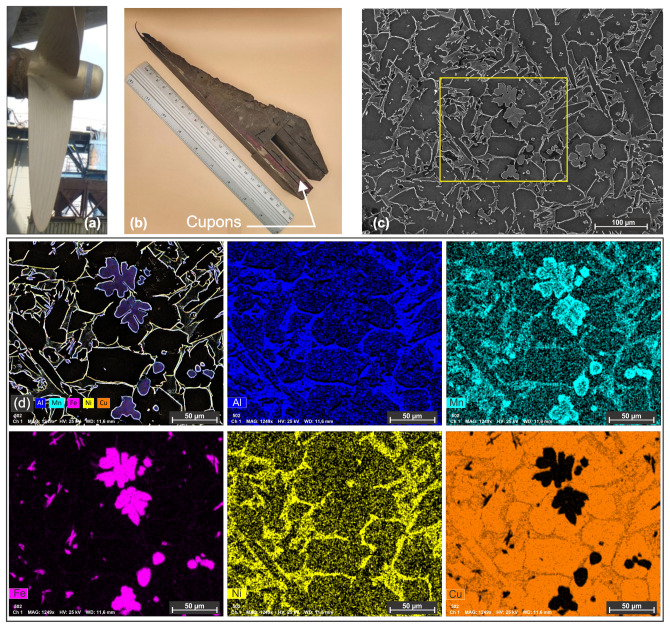
(**a**) Example of a reconditioned marine propeller from a bulk carrier vessel, (**b**) severely worn section of a marine propeller, (**c**) secondary electron micrograph of the NAB alloy extracted from the worn propeller; (**d**) EDS mapping of the NAB.

To better understand the behavior of marine the propeller material under laser cladding reconditioning techniques, aimed at maintaining the vessels seaworthiness, nickel–aluminum–bronze (NAB) was used as a base material in this study. The NAB material was extracted by cutting from a heavily worn marine propeller of a bulk carrier-type vessel. Coupons with dimensions of 100 × 25 × 8 mm were cut by Wire Electrical Discharge Machining (WEDM) as depicted in Figure 1b.

According to the ClassNK Rules for the copper alloy casting, NAB propellers are categorized into four distinct alloy types, as presented in Part K, Chapter 7 of this classification standard [27]. In this study, a KAℓBC3 alloy from the ClassNK classifications was used, with a chemical composition as presented in Table 1. The chemical composition was determined by using an energy dispersive X-ray Spectroscopy analyzer from Bruker, XFlash Detector 630M.

This material is a compositionally complex quaternary alloy, where each element plays a significant role. NAB alloy typically consists of an α phase matrix, retained β phase and a dispersion of various kappa (κ) precipitates [28], recognized by their morphology and diffusion in the matrix as κI, κII, κIII and κIV [29].

As widely accepted in the literature, NAB alloys present very good corrosion resistance in the marine environment due to the capacity of protective surface film formation (Al_2_O_3_ and Cu_2_O) [30]. The high strength and fatigue performance make NAB one of the most appropriate materials for constructing seawater exposed components [31,32]. The properties of NAB alloys can be further enhanced by heat treatments which can directly influence the hardness and the erosion corrosion behavior [33].

The ability to customize the mechanical properties and the wear resistance while preserving excellent corrosion behavior has made NAB alloys the best option for marine or submarine propellers as well as for other critical components in the marine industry [34,35,36].

The microstructure of the base material used in this study is highlighted in Figure 1c. The material has a refined microstructure with a multitude of phases, as the material is subjected to several heat treatments after the casting process [10,37,38].

**Figure 2:**
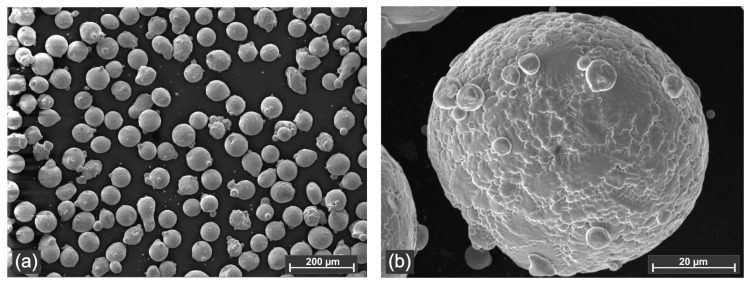
Back-scattered electron micrograph of the Metco 57 NS–Cu38Ni powder: (**a**) general appearance; (**b**) high magnification of a Cu38Ni powder particle.

NAB specimens were coated using CuNi alloyed powder with a nominal chemistry of Cu38Ni. The geometrical appearance of the powder is presented in Figure 2, with the particles being in the range of −75 + 45 μm. The Metco 57NS powder has an apparent bulk density of 3.35 g/cm^3^ and a melting temperature of 1205 °C [39].

### 2.1. Experimental Frame

To fabricate the cladding specimens, an Nd:YAG laser generator TRUMPF TruPulse 556 (Trumpf, Ditzingen, Germany) was used, together with a PRECITEC WC50 (Precitec GmbH, Gaggenau, Germany) cladding module. The optical system was calibrated to a focal distance of 200 mm and a standoff distance of 10 mm was maintained between the cladding nozzle and the substrate. The scanning speed of the laser was kept constant at 16 and 18 cm/min for all the cladding depositions with an interpass overlap of 40%. The powder was supplied into the cladding head using a Termach 1000 feeder (Termach, Appleton, WI 54914, USA) at 3.75 g/min and with a preheating temperature of 70 °C. The entire setup was operated by a 7-axis industrial robot made by CLOOS (Carl Cloos Schweißtechnik GmbH, Haiger, Germany). The samples were preheated using a digital preheating table calibrated and checked by a thermocouple.

**Figure 3:**
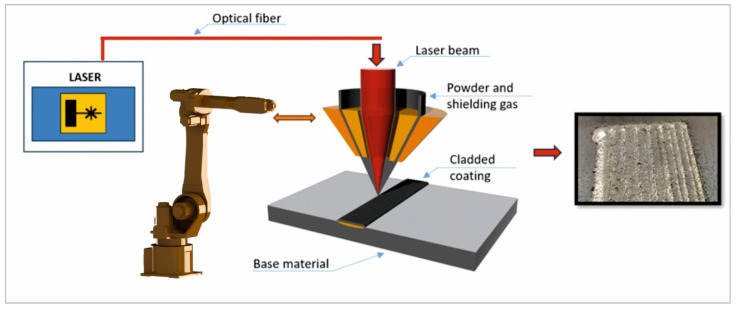
Schematic of the experimental laser cladding setup.

The samples were prepared by cutting using WEDM to prevent any thermal influence that might affect the later response of the NAB. Prior to the deposition, the substrate surface was cleaned using sandpaper (P 1200 grain size) and rinsed with ethanol to remove impurities. The schematic of the laser cladding setup is presented in Figure 3.

The laser cladding process is highly dependent on the selected processing parameters. The interaction between the laser beam and the substrate significantly affects how the powder behaves, which will influence both the geometry and mechanical behavior of the cladded layer. To evaluate these effects, as a preliminary step, single-track laser cladding tests were made to evaluate how the process parameters influence the geometry of single tracks. The constant parameters were a pulse duration of 3 ms, cladding speed of 16 cm/min and substrate preheat of 180 °C. The laser power was varied in 3 steps: 4000 W, 3800 W and 3600 W. The analysis of samples 1.1, 1.2 and 1.3 revealed that laser power is an important factor in obtaining defect free claddings with moderate dilution with the substate. The tests were used to establish the clad width needed for calculating the degree of cladding overlap.

**Figure 4:**
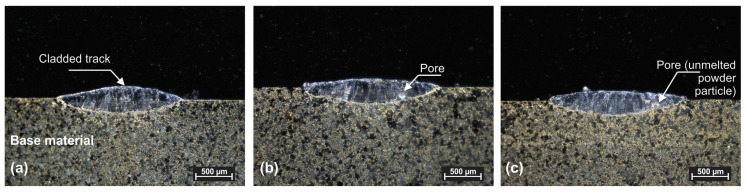
Optical macroscopy for single-pass initial laser cladding tracks. (**a**) Sample 1.1, (**b**) sample 1.2 and (**c**) sample 1.3.

As shown in Figure 4, cladding of Cu38Ni powder onto an NAB substrate presents certain challenges, particularly in obtaining low dilution and avoiding pores and crack formation. Based on these results, an optimal parameter window for single tracks was identified: 4000 W of laser power with a repetition rate of 42 Hz, combined with mandatory substrate preheating to at least 180 °C.

For the second step, partially overlapped tracks were fabricated. The parameters were adjusted accordingly, as presented in Table 2. To compensate for the heat accumulation during the long processing time, both laser power and preheating temperature were slightly reduced. For the same reason, the cladding speed was increased from 16 to 18 cm/min.

### 2.2. Methods

The microstructure of the samples was examined using an inverted optical microscope (Leica DMILM LED by Leica Microsystems GmbH, Wetzlar, Germany) and a scanning electron microscope (TESCAN VEGA LMU, TESCAN Group, Brno, Czech Republic) equipped with an EDS detector Bruker XFlash 630M (Bruker Nano GmbH, Berlin, Germany).

Microhardness measurements of the cladded layers were carried out with an INNOVATEST Falcon 600G2 (INNOVATEST Europe BV, Maastricht, The Netherlands) hardness tester, capable of loads from 0.1 g to 62 kg. The Vickers method was used with a 200 g load and a dwell time of 10 s. For each analyzed region, five indentations were performed, and the average value was considered representative.

The electrochemical characterization was performed using a conventional three-electrode cell model and an SP-150 potentiostat/galvanostat (Biologic, Seyssinet-Pariset, France). The working electrode was the investigated sample, with a 1 cm^2^ surface area exposed to the electrolyte. A platinum wire served as the counter electrode, and a saturated calomel electrode (SCE) was used as the reference. The measurements were conducted in a 3.5 wt.% NaCl solution, selected to simulate a marine environment. Linear polarization resistance (LPR) tests were carried out at a scan rate of 0.001 V/s to determine the corrosion potential and polarization behavior of the samples. Three tests were made on each specimen and the Tafel plots were analyzed using the PSTrace software with the Butler–Volmer fitting equation to determine the potential (E_corr_) and corrosion current density (I_corr_).

## 3. Results and Discussion

**Figure 5:**
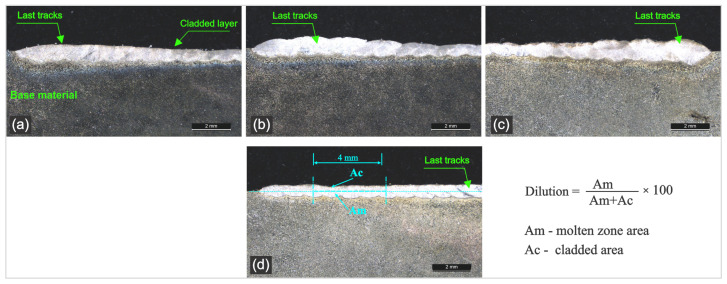
Optical microscopy of cladded layer cross-section: (**a**) sample 1, (**b**) sample 2, (**c**) sample 3 and (**d**) sample 4.

The coating morphology of multi-pass cladded layers was analyzed in cross-sections using optical microscopy as presented in Figure 5.

The analysis of the cross-sectional micrographs revealed a generally uniform and continuous deposition profile over the entire cladded area. The interface between adjacent tracks is clearly defined indicating good overlapping during the laser cladding process. It can be observed that the geometrical profile is slightly different at the last tracks. Multiple tracks have been applied and the cumulative heat input led to a different thermal behavior of the last tracks. A gradual increase in clad height and dilution was observed toward the final tracks of each specimen. It is a common challenge in laser cladding. The thermal behavior in case of the laser tracks is different, as the cooling rate of the last tracks is slower, which extends the solidification time and increases the dilution rate but also affects the layer geometry.

Despite the different thermal gradients, the quality of the cladded tracks is high with no visible cracks and good structural integrity and good adhesion to the substrate. The absence of defects validated the determined process parameters for pulsed laser cladding even if there are variations in the thermal conditions across the sample.

The surface of the coating shows several unmelted or partially melted particles that are common in powder feed laser cladding associated with the process complexity.

The presence of these superficial irregularities is not considered crucial as the external surfaces of the cladded layers are normally subjected to a machining process, which removes any surface roughness.

### 3.1. Microstructure

The microstructure of the cladded samples was analyzed in cross-sections using optical microscopy and SEM, after standard metallographic preparation and etching. High density coatings with only few pores and no cracks have been obtained through pulsed laser deposition. The cladded layer has a heterogeneous structure, consisting of columnar dendrites and fine equiaxed grains.

Despite the application of preheating to reduce thermal stress, the presence of coarse dendritic structure near the interface with the substrate indicates that the cooling rate in this region remains high enough to promote directional solidifications. This microstructural pattern is observed in all four samples, with more pronounced dendritic growth in samples 1 and 2. In the central and upper regions of the layer, the microstructure is formed by equiaxed grains.

The formation of a finer and uniformly distributed structure in these regions is influenced by the localized thermal field generated by the previous cladded track, which acts as a supplementary preheat source and promotes a more uniform temperature distribution. The slower cooling rate in those regions promotes nucleation over directional growth.

**Figure 6:**
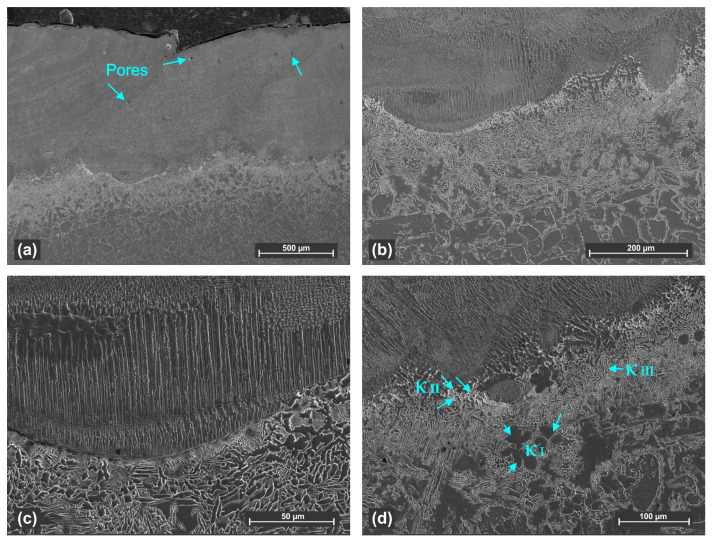
Secondary electron micrograph of sample 1: (**a**) general appearance of the cross-section; (**b**) detail of the coating interface with the NAB substrate; (**c**,**d**) microstructural details of the fusion line.

Figure 6 shows the cross-section of sample 1, with details of the interface zone and of the heat effected zone of the substrate (HAZ). The cladded layer is dense and compact with only three small pores observed at the overlapping area between tracks. The pores are isolated and observed only in sample 1. The microstructure near the substrate mainly consists of large dendrites with a clear well defined fusion line between materials as shown in Figure 6c or by an uneven boundary between coating and substrate as visible in Figure 6d. In both cases, the interface region towards the substrate contains numerous iron-rich κI precipitates and hard phases rich in aluminum that indicate rapid solidification near the fusion line.

**Figure 7:**
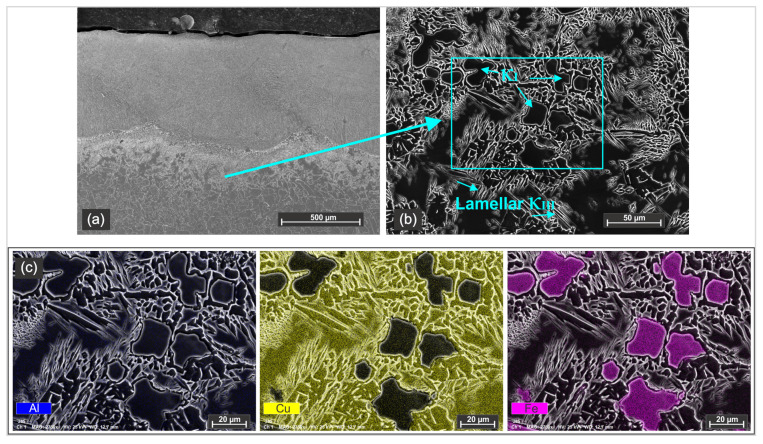
Secondary electron micrograph and EDS of sample 2: (**a**) general appearance, (**b**) detail of the microstructure of the heat affected zone and (**c**) EDS mapping of the NAB substrate showing the aluminum, copper and iron distribution in the area from micrograph (**b**).

The cress section of sample 2 is shown in Figure 7. The structure is similar to that of sample 1, showing the same specific microstructure of the coating and comparable distribution of the phases within the HAZ. As in sample 1, iron-rich precipitates embedded in a copper-rich matrix were identified in the heat-affected zone.

Figure 7c shows the EDS mapping of this area highlighting the distribution of elements and confirming the formation of the Fe-rich κI phases.

Nickel–aluminum–bronze (NAB) alloys subjected to heat treatments or rapid heating–cooling cycles can form various phases depending on chemical composition and solidification time. Laser cladding is a very fast processing technique and rapid cooling of the substrate in the HAZ often results in non-equilibrium solidification conditions. Therefore, multiple phases typical of NAB α/β and four types of κ phases can coexist.

**Figure 8:**
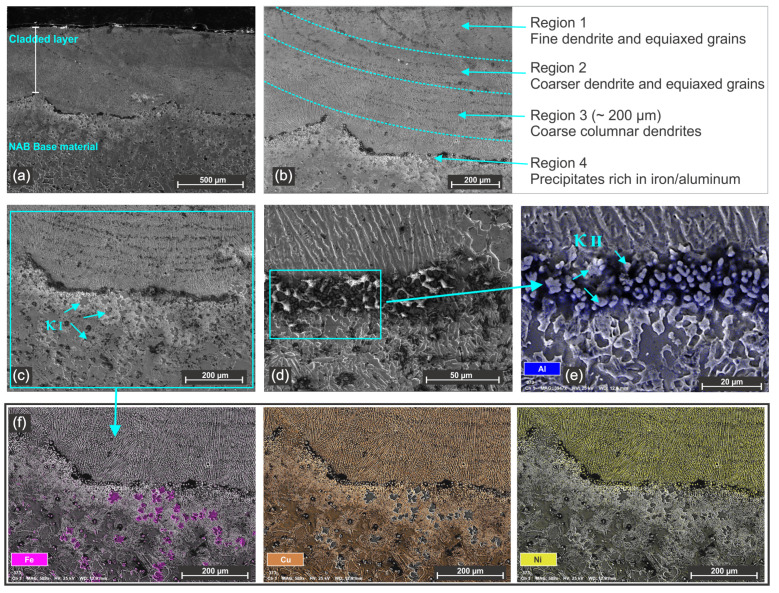
Secondary electron micrograph and EDS of sample 4: (**a**) general appearance, (**b**) detail of the different microstructure, (**c**) interface and HAZ area and indication of κI phases, (**d**) interface fusion line between the coating and substrate, (**e**) EDS mapping of interface fusion line and (**f**) EDS mapping indicating the Fe, Cu and Ni distribution according to area from micrograph (**c**).

Figure 7b illustrates the coexistence of at least two κ phases in the HAZ, where the temperature range can vary between 1100 °C and 500 °C during processing. The κI phase is easily identifiable due to the larger size, distinct morphology and high iron content, as κI phases typically form only if more than 5 wt% Fe is present. The position near the subsurface of the substrate indicates that, due to the high thermal gradient, diffusion of iron occurred from the base material toward this area. The formation of these phases is also highlighted in Figure 8c,f.

The presence of the lamellar κIII phase was observed in the HAZ of all investigated samples. This phase forms under rapid thermal cycles and localized aluminum segregation during solidification. Sample 3 shows the same morphological appearance as sample 2 with no pores or defects. Significant differences appear in sample 4, which was produced with the highest laser power among the four samples. This sample exhibits the highest percentage of dilution, approximately 48%. A high percentage of dilution generates a good metallurgical bonding with the substrate and it also promotes element diffusion from the substrate, which can have a major influence on the mechanical properties of the coating and on the corrosion resistance.

Figure 8 shows the microscopy and EDS mapping of sample 4, where the large number of iron-rich phases within the HAZ can be clearly seen. Additionally, the interface region between the coating and the substrate shows the formation of aluminum rich precipitates κII phases, aligned along the fusion line. This sample was fabricated with the highest laser energy (11.2 J) and 175 °C preheating, which led to a uniform cladded layer that was defect-free but with high dilution and reduced mechanical properties.

The microscopy analyses reveal that the process and parameters of the pulsed laser are effective in fabricating CuNi layers on an NAB substrate with dense coatings with uniform morphology and good bonding with the substrate. The analysis supports the suitability of the pulsed laser cladding for reconditioning NAB marine components.

### 3.2. Microhardness

Vickers microhardness testing was performed on all samples to determine the mechanical behavior of the cladded layers. The measurements were carried out on three distinct regions of the coating, starting from the top of the layers and continuing through the middle and lower zones as shown in Figure 8b. For comparison, the interface zone and the NAB substrate were also tested.

**Figure 9:**
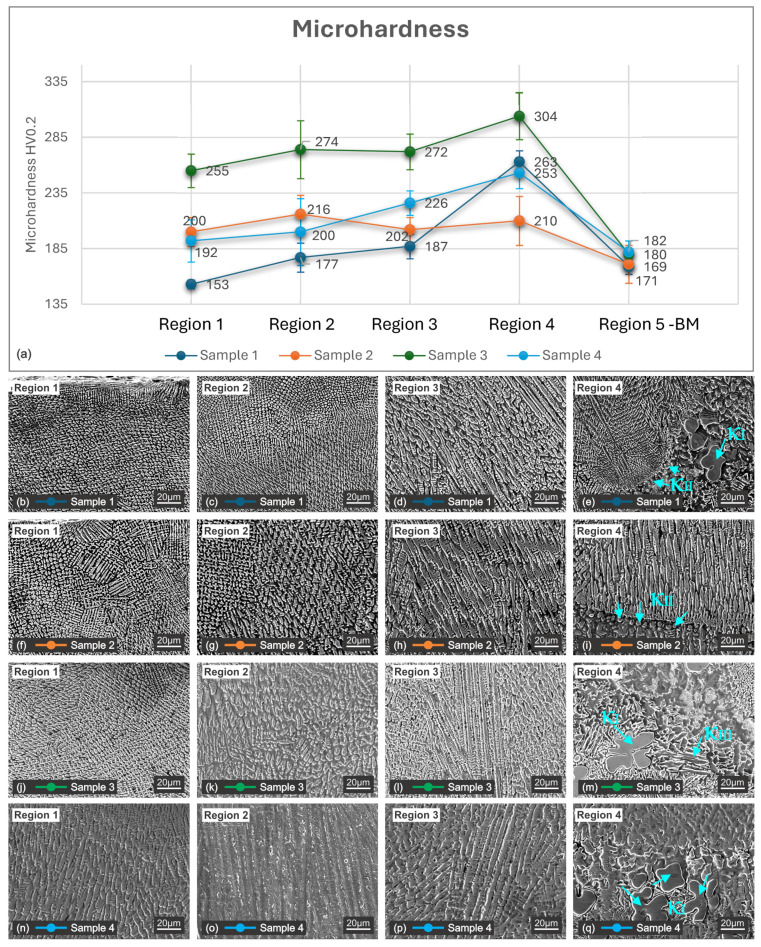
Microhardness of the 4 samples: (**a**) microhardness profile over the 4 regions that have been measured; (**b**–**q**) representative microstructures associated with each area of microhardness testing.

The results indicate that laser power has a major influence on the microhardness of the cladded layers. Figure 9 presents the microhardness profile across the cladded thickness and representative microstructures of each region. As illustrated, an increase in laser power increases the microhardness up to a certain point, after which it decreases in all four measured zones. This behavior is associated with the energy input, which influences the solidification rate, grain refinement, phase formation and the diffusion rate.

It can be observed that the highest microhardness is recorded in sample 3, reaching 304 HV0.2, followed by sample 4. In sample 4, excessive dilution shifts the coating composition closer to that of the NAB substrate, reducing the proportion of the strengthening CuNi matrix and leading to a reduction in the coating’s microhardness. These results confirm that a minimum amount of energy is essential to obtain a dense, defect-free coating, but excessive diffusion and dilution should be avoided. Notable is the distribution of hardness over the different zones on the cross-section. Region 1 (top of the layer) consists primarily of Cu-Ni solid solution with short columnar dendrites (3–20 µm) in the case of samples 1, 2 and 3. Sample 4 is distinguished by larger dendrites with dimensions up to 20 µm (Figure 9n). In all four samples, diffusion from the substrate is present but limited, as shown in Table 3. This region exhibits the lowest hardness across the coating thickness. It is unusual because a finer dendrite arm spacing normally increases hardness, but in this case, the microhardness is not influenced mainly by the dendrite size, since the composition (aluminum and iron content) plays an important role in strengthening the CuNi matrix.

Region 2 presents a transition microstructure with coarser dendrites and equiaxed grains. In this region, the influence of the laser power is clearly visible as the dendrite structure increases from 3 to 5 µm in the case of sample 1 (Figure 9c) to 20 µm in the case of sample 4 (Figure 9o).

Region 3, the thickest area (~200 µm), shows large dendrites oriented towards the thermal field (Figure 9d,h,i,p). Dendrites of 100 µm are visible in this region on all four samples.

The highest hardness is measured in region 4 near the interface with the base material (~250–304 HV0.2). In this area, due to the strong thermal gradient, the structure is formed mainly by coarse dendrite structures enriched in Fe/Al that promote the k-phase precipitation along the interface line, as shown in Figure 9e,m,q. According to Table 3, in this region, the aluminum and iron diffusion is at its maximum, with up to 5.20% aluminum and 3.78 iron in the case of sample 3. Moreover, in this area and in the HAZ, the presence of lamellar κIII (Figure 9m) further contributes to increasing hardness.

Similar observations have been reported by Zhao et al. [40] in the case of NAB cladding on a stainless-steel substrate.

The diffusion of aluminum and iron across the interface influences the hardness variations. Overall, the results validate that pulsed laser cladding ensures strong adhesion between the cladded layer and substrate, providing improved mechanical properties, especially in the transition zone. On average, the hardness measured in the middle of the layer is approximately 100 Vickers units higher than the NAB base material.

### 3.3. Corrosion Resistance

The corrosion resistance was determined on the top surface of the cladded samples. To ensure consistent evaluation of the corrosion behavior, approximately one-third of the top layer of the laser-cladded coatings was removed prior to testing. The exposed surface was then successively ground and polished using SiC abrasive papers up to 2000 grit to obtain a uniform finish.

**Figure 10:**
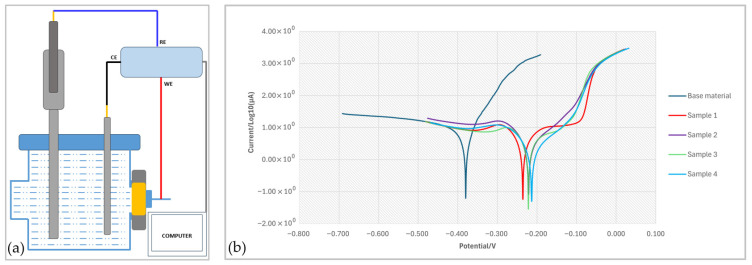
(**a**) Schematic diagram of the conventional three-electrode electrochemical cell (CE is the counter electrode, RE the reference electrode and WE the working electrode); (**b**) linear polarization curves in 3.5% NaCl solution for samples 1–4 and the NAB substrate.

Figure 10 shows the experimental setup used for corrosion determination and in Figure 10b, the linear polarization curves for the substrate and for the samples 1 to 4 are presented. It can be clearly seen that corrosion behavior is better for the coated samples compared with the NAB base material. In the case of the base material, the current continues to increase steadily with potential, indicating the absence of a passive film on the surface and ongoing corrosion. In contrast, samples 1 to 4 have a lower current over the potential range, especially below −0.25 V, indicating a lower corrosion rate and a partial passivation of the surface. Sample 4 is characterized by the best corrosion resistance, 0.10 mm/year, due to the passive region between −0.20 and −0.17 V, which indicates the formation of a stable passive film, likely composed of complex nickel, copper and aluminum oxides. Sample 4 also has the highest content of aluminum and iron diffused from the substrate. The increased aluminum content in region 2 promotes the formation of an alumina-rich passive film that enhances corrosion resistance. Iron also contributes to passivation by stabilizing the oxide film. Good corrosion resistance, with low current densities, is also obtained for sample 3, which indicates the formation of a passive, stable and protective layer of oxides.

The results show that there is a clear dependence between the microstructure, diffusion and corrosion resistance. The corrosion resistance of all four coated samples is higher than the NAB base material as presented in Table 4.

Samples 3 and 4 realized with a higher laser energy benefit from the formation of a dense microstructure with increased hardness and enhanced corrosion resistance due to the formation of intermetallic phases.

## 4. Conclusions

This study demonstrates the applicability of the pulsed laser cladding for enhancing NAB alloys or for reconditioning NAB components by using a CuNi alloy powder. The process is highly dependent on the setup and parameters, but after optimization, it can produce defect-free cladded layers, with good metallurgical bonding and enhanced mechanical characteristics and improved corrosion resistance.

The microstructural analyses revealed a combination of columnar and equiaxed grains in the coating, along with k-phase precipitates (κI–κIII) at the interface with the base material, influenced by the thermal effect and dilution. Overall, sample 3, produced with a higher laser energy density, achieved the highest microhardness of 304 HV02 and a reduced corrosion rate of approximately 0.16 mm/year compared with the base material. Sample 4, despite the slightly lower microhardness (263 HV02), is characterized by the best corrosion resistance at approximately 0.10 mm/year, indicating the good balance between mechanical and corrosion resistance properties.

A key factor in laser cladding of Cu38Ni on NAB substrate is the dilution, which ranged from moderate to high (48%), with higher values improving bonding but reducing the hardness due to the increased element diffusion. However, a higher dilution also promotes the formation of complex phases that may limit chloride penetration and enhance corrosion resistance. The optimal parameters for pulsed laser cladding of CuNi on an NAB substrate are pulse energy of 10.8 J, pulse duration of 3 ms at a repetition rate of 42 Hz and substrate preheating at 165 °C. Using this setup, high-quality coatings with enhanced hardness and significantly better corrosion resistance compared with the NAB substrate were obtained; further research will focus on evaluating the cavitation erosion resistance and applying this technique to repair an actual damaged propeller.

## Figures and Tables

**Table 1:** Chemical composition of the base material and powder.

	Element Wt. (%)				
	Al	Mn	Fe	Cu	Ni
Substrate KAℓBC3 *	5.49	3.55	4.15	80.80	6.00
Powder Metco 57NS	-	-	-	62.00	38.00

* Composition determined by EDS, Bruker X Flash detector 630 m, 5 determinations on 2 × 2 mm area. Standard deviation is 0.22 for Cu, Ni, Fe, Mn and 0.57 for Al.

**Table 2:** Parameters of laser cladding.

Sample	Laser Power [W]	Pulse Energy [J]	Pulse Duration [ms]	Frequency [Hz]	Preheat [°C]	HAZ Depth [µm]
1	3200	9.6	3	42	165	195
2	3400	10.2	3	42	165	250
3	3600	10.8	3	42	165	340
4	3800	11.2	3	42	165	372

**Table 3:** EDS measurements of aluminum and iron content in regions 1 to 4 of cladded samples.

Region/Sample	Sample 1	Sample 2	Sample 3	Sample 4
**Region 1** Element wt. (%)	2.51Al 1.59Fe	2.59Al 1.67Fe	3.50Al 1.36Fe	3.35Al 1.95Fe
**Region 2** Element wt. (%)	2.73Al 1.55Fe	2.55Al 1.58Fe	3.35Al 1.37Fe	3.58Al 2.07Fe
**Region 3** Element wt. (%)	3.09Al 1.74Fe	2.66Al 1.46Fe	3.29Al 1.43Fe	3.40Al 2.25Fe
**Region 4** Element wt. (%)	5.20Al 3.81Fe	5.14Al 3.78Fe	5.07Al 1.88Fe	5.16Al 2.29Fe

**Table 4:** Corrosion rate of samples 1 to 4 and NAB substrate (n = 3).

Sample	E_corr_ [V]	I_corr_ [µA]	Corr. Rate [mm/Year]
**Substrate**	−0.380 ± 0.03	18.141 ± 1.107	0.58 ± 0.03
**Sample 1**	−0.235 ± 0.03	9.442 ± 0.855	0.30 ± 0.04
**Sample 2**	−0.211 ± 0.03	7.423 ± 0.696	0.24 ± 0.03
**Sample 3**	−0.222 ± 0.04	5.117 ± 0.879	0.16 ± 0.03
**Sample 4**	−0.213 ± 0.04	3.383 ± 0.604	0.10 ± 0.04

## Data Availability

The original contributions presented in this study are included in the article material. Further inquiries can be directed to the corresponding author.
